# Efficacy and pharmacokinetics of ozoralizumab, an anti-TNFα NANOBODY^®^ compound, in patients with rheumatoid arthritis: 52-week results from the OHZORA and NATSUZORA trials

**DOI:** 10.1186/s13075-023-03036-4

**Published:** 2023-04-13

**Authors:** Tsutomu Takeuchi, Yukihiro Chino, Masafumi Kawanishi, Megumi Nakanishi, Hirotaka Watase, Yoko Mano, Yuri Sato, Saeko Uchida, Yoshiya Tanaka

**Affiliations:** 1 Keio University School of Medicine, Tokyo, Japan; 2 Saitama Medical University, Saitama, Japan; 3 Taisho Pharmaceutical Co., Ltd., Tokyo, Japan; 4 University of Occupational and Environmental Health, Japan, Kitakyushu, Japan

**Keywords:** Pharmacokinetics, Pharmacodynamics, Arthritis rheumatoid, Tumor necrosis factor alpha inhibitor, Antirheumatic agent, Ozoralizumab

## Abstract

**Introduction:**

Ozoralizumab (OZR), a tumor necrosis factor alpha (TNFα) inhibitor, is a NANOBODY^®^ compound that binds to TNFα and human serum albumin. The main objective of this study was to analyze the pharmacokinetics (PK) of the drug and its correlation with clinical efficacy in patients with rheumatoid arthritis (RA).

**Methods:**

Efficacy data were analyzed from the OHZORA trial, in which OZR 30 or 80 mg was administered to Japanese patients with RA at 4-week intervals for 52 weeks in combination with methotrexate (MTX; *n* = 381), and the NATSUZORA trial, in which OZR 30 or 80 mg was administered without concomitant MTX (*n* = 140). Effects of patient baseline characteristics and anti-drug antibodies (ADAs) on the PK and efficacy of OZR were investigated, and a post hoc analysis of PK effects on drug efficacy was performed.

**Results:**

The maximum plasma concentration (C_max_) was reached in 6 days in both the 30 and 80 mg groups, with an elimination half-life of 18 days. The C_max_ and area under the plasma concentration–time curve increased in a dose-dependent manner, and the trough concentration reached steady state by week 16. The exposure of OZR correlated negatively with patient body weight and was not affected by other patient baseline characteristics. Effects of ADAs on the exposure and efficacy of OZR were limited in both trials. However, antibodies that neutralize the binding to TNFα had some effect on the exposure and efficacy of OZR in the NATSUZORA trial. The receiver operating characteristic analysis of the effect of trough concentration on the American College of Rheumatology 20% and 50% improvement rates was retrospectively performed, and a cutoff trough concentration of approximately 1 μg/mL at week 16 was obtained in both trials. The efficacy indicators in the subgroup with trough concentration ≥ 1 μg/mL were higher than those in the < 1 μg/mL subgroup at week 16, while no clear cutoff was obtained at week 52 in both trials.

**Conclusions:**

OZR showed a long half-life and favorable PK properties. A post hoc analysis suggested sustained efficacy independent of trough concentration by subcutaneous administration of OZR 30 mg at 4-week intervals for 52 weeks.

**Trial registration:**

JapicCTI, OHZORA trial: JapicCTI-184029, registration date July 9, 2018; NATSUZORA trial: JapicCTI-184031, registration date July 9, 2018.

**Supplementary Information:**

The online version contains supplementary material available at 10.1186/s13075-023-03036-4.

## Background

Rheumatoid arthritis (RA) is a disease in which persistent inflammation based on an autoimmune response develops in multiple joints, leading to destructive arthritis [1]. Methotrexate (MTX) is one of the first-line pharmacotherapeutic agents used to treat RA. If treatment goals are not achieved or the use of MTX is not feasible, use of agents such as conventional synthetic disease-modifying antirheumatic drugs (csDMARDs), biologic DMARDs (bDMARDs), and Janus-associated kinase (JAK) inhibitors is recommended [2, 3]. Tumor necrosis factor alpha (TNFα) plays a significant role in the pathogenesis of RA [4]. TNF inhibitors were one of the first bDMARDs developed to treat RA and hold an important position in the pharmacotherapy of RA [5–8].

Ozoralizumab (OZR) is a next-generation TNFα inhibitor with a bispecific structure linking two humanized anti–TNFα NANOBODY^®^ VHHs with a humanized anti–human serum albumin (HSA) NANOBODY^®^ VHH [9, 10]. NANOBODY^®^ VHHs are the variable regions of heavy-chain-only antibodies derived from camelids. VHH domains of 12–30 kDa size have excellent stability, have the ability to bind to targets that cannot be reached by conventional antibodies of approximately 150 kDa, may be less immunogenic, are modular, and are expected to be produced quickly at low cost [11–13]. As it is composed of NANOBODY^®^ VHHs, OZR is relatively small, with a molecular weight of 38 kDa. Mouse experiments have demonstrated that its conjugation with an anti-HSA NANOBODY^®^ VHH enhances localization to inflamed joints, reduces excretion through the kidneys, and increases its retention in the blood [9].

In the OHZORA trial, the efficacy, safety, and pharmacokinetics (PK) of OZR were investigated with OZR 30 or 80 mg being administered subcutaneously at 4-week intervals in combination with MTX to Japanese patients with RA who had active disease despite being treated with MTX. The results showed significant improvement in clinical symptoms with both 30 mg and 80 mg of OZR compared with placebo from day 3 to week 24 of administration [10], and the efficacy was maintained until week 52 [14]. In the NATSUZORA trial, the efficacy, safety, and PK of OZR were investigated with OZR 30 or 80 mg being administered subcutaneously at 4-week intervals for 52 weeks without MTX to patients with active RA who had inadequate response or were intolerant to prior csDMARDs. Improvement in clinical symptoms was observed from 1 week after administration, and the effects were maintained until week 52 [15].

In the present study, we investigated the PK profile and the effects of patient baseline characteristics and anti-drug antibodies (ADAs) on the PK and efficacy of OZR in Japanese patients with RA from the OHZORA and NATSUZORA trials. We also assessed the relationship between the trough concentration of OZR and efficacy by post hoc analysis.

## Methods

### Study design and population

Data analyzed in this study are from the OHZORA trial, conducted between September 2018 and October 2020 [10, 14], and the NATSUZORA trial, conducted between October 2018 and October 2020 [15]. Both studies were multicenter, randomized trials conducted in Japanese patients with RA to evaluate the efficacy and safety of a 52-week OZR administration. The OHZORA trial consisted of two periods: period A, which was a 24-week, placebo-controlled, double-blind period, and period B, which was a 28-week, open-label period. OZR 30 or 80 mg was administered subcutaneously at 4-week intervals in combination with MTX. The OHZORA trial population included patients with active RA who had an inadequate response to MTX. Patients were required to be taking MTX at least 12 weeks before baseline, and their doses (6–16 mg/week) should not have been changed within 6 weeks before baseline. In period A, patients were randomly assigned to receive OZR 30 mg, OZR 80 mg, or placebo. If patients in the placebo and 30 mg groups had less than 20% improvement from baseline in the tender joint count in 68 joints (TJC68) and swollen joint count in 66 joints (SJC66) at week 16, they were transitioned to the 30 mg and 80 mg groups, respectively, at week 20 (early escape [EE]). The remaining patients from the placebo group were randomly reassigned to the 30 mg or 80 mg groups at week 24 and entered period B. The dose of MTX was 6–16 mg/week for both periods. In the NATSUZORA trial, OZR 30 or 80 mg was administered subcutaneously at 4-week intervals without MTX open-label. Patients in the NATSUZORA trial were those with active RA who had used csDMARDs, including MTX, at least from 12 weeks before baseline to 4 weeks before baseline or who had discontinued csDMARDs, including MTX, due to safety concerns.

The full analysis set (FAS) comprised patients who had received at least one dose of the study drug and in whom at least one efficacy endpoint was observed after receiving the study drug. The PK analysis set was defined as a subset of the FAS from which patients with insufficient or overdosing volume were excluded. For the period A (until week 24) evaluation in the OHZORA trial alone, patients with less than two-thirds of the planned number of administrations were excluded.

### Sample collection and PK analysis

In all patients, trough concentrations were measured before administration; at 4, 8, 16, 20 (EE only), 24, 40, and 52 weeks after administration; at the follow-up examination; and at the time of discontinuation in the OHZORA trial and before administration; at 4, 8, 16, 24, and 52 weeks after administration; at the follow-up examination; and at the time of discontinuation in the NATSUZORA trial. Plasma OZR concentrations were measured using a validated enzyme-linked immunosorbent assay (ELISA). OZR in the sample was captured on a TNFα-immobilized 96-well plate. Rabbit anti-OZR polyclonal antibody, horseradish peroxidase–conjugated goat anti-rabbit IgG, and 3,3′,5,5′-tetramethylbenzidine were added, and the absorbance at 450 nm was measured with a microplate reader. The lower limit of quantification in this assay was 0.1 μg/mL. In the OHZORA trial, in addition to the time points described above, plasma OZR concentrations were measured at 2 days and 1, 2, and 3 weeks after the first drug administration in 213 patients from whom consent was obtained (placebo, 46; OZR 30 mg, 82; OZR 80 mg, 85), and the maximum plasma concentration (C_max_), time to reach the C_max_ (t_max_), area under the plasma concentration–time curve (AUC), elimination half-life (t_1/2_), apparent clearance (CL/F), and apparent volume of distribution (V_d_/F) were calculated from the data until 4 weeks after the first administration using noncompartmental analysis. The mean plasma OZR concentration was simulated by fitting it using a compartment model including CL/F, first-order absorption rate constant (K_a_), and V_d_/F. For the noncompartmental analysis and simulation, Phoenix WinNonlin version 8.0 (Certara, Princeton, NJ, USA) was used.

### Immunogenicity assessment

Plasma ADAs were measured before administration; at 8, 20 (EE only), 24, 40, and 52 weeks after administration; at the follow-up examination; and at the time of discontinuation in the OHZORA trial and before administration; at 8, 24, and 52 weeks after administration; at the follow-up examination, and at the time of discontinuation in the NATSUZORA trial. ADAs were measured by a multi-tiered testing approach involving two stages, a screening assay and a confirmatory assay [16], using a validated electrochemiluminescence immunoassay that incorporates an acid dissociation treatment. ADA evaluation was classified as negative, positive before administration without an increase in antibody titer after administration (baseline [BL]-positive), positive before administration with an increase in antibody titer after administration (treatment-boosted [TB]–positive), or positive after administration (treatment-induced [TI]–positive). Neutralizing antibodies (NAbs) were measured using a validated competitive ELISA that incorporated an acid dissociation treatment for ADA-positive plasma samples. Patients indicated as positive for NAbs at least once in all measurements were classified to be NAb-positive. The drug tolerance of these methods (permissible drug concentration in the samples) was ≤ 10 μg/mL of OZR.

### Statistical analysis

Baseline demographic and clinical characteristics were analyzed in the FAS. Correlation coefficients among patient baseline characteristics, such as age, body weight, disease duration, serum albumin concentration, estimated glomerular filtration rate, MTX dose at the start of the drug administration, high-sensitivity C-reactive protein (hs-CRP), erythrocyte sedimentation rate (ESR), and disease activity and trough concentration at week 4 or the C_max_ or AUC from 0 to infinity (AUC_0-∞_) after the first administration, were calculated in the PK analysis set. Effects of patient baseline characteristics on the 20% improvement according to the American College of Rheumatology criteria (ACR20) response rate at week 16 or week 24 in the OHZORA or NATSUZORA trials, respectively, were investigated by subgroup analysis of age, gender, body weight, hs-CRP, Disease Activity Score in 28 joints based on CRP (DAS28-CRP), Disease Activity Score in 28 joints based on ESR (DAS28-ESR), Clinical Disease Activity Index (CDAI), Simplified Disease Activity Index (SDAI), previous use of bDMARDs, the number of TNFα inhibitors used, seropositivity, ADAs, and NAbs in the FAS. Effects of seropositivity, ADAs, or NAbs on the plasma concentrations of OZR were investigated in the PK analysis set. To investigate the effects of the trough concentration on the ACR20 response rate in the OHZORA (period A) and NATSUZORA trials, we compared the four quantiles that were divided by quartile points of plasma OZR trough concentration at week 16 calculated using SAS software version 9.4 (SAS Institute, Tokyo, Japan) in the PK analysis set. We also compared the segment below the first quartile point with each of the segments equal to and above the first quartile point in the 30 mg + 80 mg or 30 mg groups.

Post hoc analysis was performed with the PK analysis sets in both trials. For the OHZORA trial, the data after dose changes due to EE were excluded from the PK analysis set. Receiver operating characteristic (ROC) analysis of the relationship between ACR20 or 50% improvement according to the American College of Rheumatology criteria (ACR50) response rates and trough concentrations at weeks 16, 24, or 52 was performed to estimate cutoff values for trough concentration. Cutoff values were selected by the Youden index and adopted if the AUC of the ROC curve was > 0.5, 95% confidence interval (CI) of the AUC was > 0.5, and sensitivity at the cutoff value was ≥ 0.8. At time points where a cutoff value for the trough concentration was obtained, the effect of the cutoff value on efficacy was investigated by comparing a subgroup with trough concentration equal to or above the cutoff value and a subgroup with trough concentration below the cutoff value using Fisher’s exact test for each 30 mg group in both trials.

ROC analysis was performed using IBM SPSS Statistics 27 (IBM, Armonk, NY, USA), and Fisher’s exact test was performed using SAS software version 9.4. *p*-values less than 0.05 were considered to be statistically significant.

## Results

### Patient characteristics and baseline demographics

**Fig. 1:**
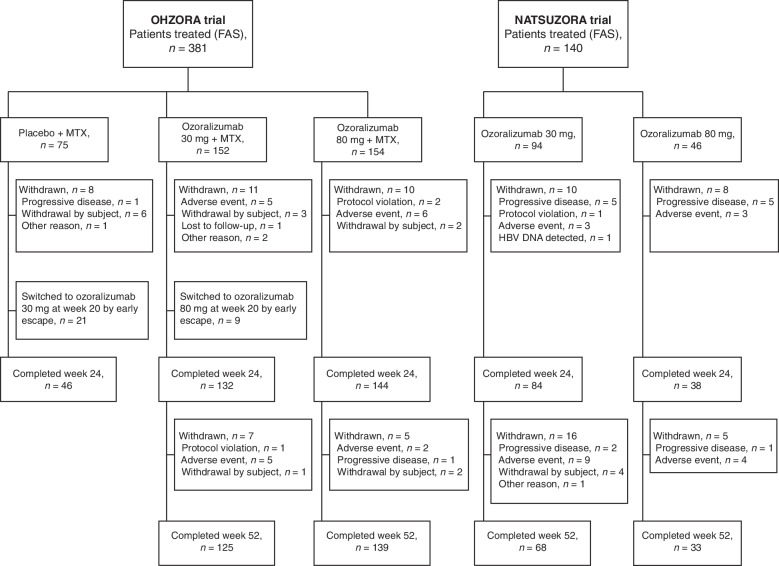
Flowchart of patient disposition. *FAS* full analysis set, *HBV DNA* hepatitis B virus DNA, *MTX* methotrexate

In the OHZORA trial, the FAS included 381 patients (placebo, 75; OZR 30 mg, 152; OZR 80 mg, 154), while the PK analysis set included 380 patients in total (minus one patient with insufficient dosing volume; placebo, 75; OZR 30 mg, 152; OZR 80 mg, 153) and 362 patients in period A (minus 18 patients with insufficient number of administrations; placebo, 69; OZR 30 mg, 147; OZR 80 mg, 146). The FAS and PK analysis set of the NATSUZORA trial were the same population and included 140 patients (OZR 30 mg, 94; OZR 80 mg, 46) (Fig. 1). In each study, no notable differences were observed in the mean age, MTX dose, disease activity, extent of joint damage, and physical function, among other characteristics, between the treatment groups (Table 1).

**Table 1:** Baseline demographic and clinical characteristics of patients

**Characteristic**	**OHZORA trial**			**NATSUZORA trial**	
	**Placebo + MTX (*n* = 75)**	**Ozoralizumab 30 mg + MTX (*n* = 152)**	**Ozoralizumab 80 mg + MTX (*n* = 154)**	**Ozoralizumab 30 mg (*n* = 94)**	**Ozoralizumab 80 mg (*n* = 46)**
Age, years	54.3 ± 12.1	54.8 ± 11.2	55.5 ± 10.9	58.0 ± 12.3	57.6 ± 13.1
< 65 years, n (%)	56 (74.7)	119 (78.3)	116 (75.3)	59 (62.8)	28 (60.9)
Female, n (%)	57 (76.0)	105 (69.1)	123 (79.9)	71 (75.5)	40 (87.0)
Body weight, kg	58.4 ± 13.5	60.0 ± 12.8	57.6 ± 11.6	59.7 ± 14.6	59.0 ± 12.7
Disease duration, years	7.6 ± 7.4	6.8 ± 6.4	7.8 ± 7.5	7.0 ± 7.4	10.0 ± 10.2
Serum albumin, g/dL	3.9 ± 0.3	3.9 ± 0.3	3.9 ± 0.3	3.8 ± 0.3	3.8 ± 0.4
eGFR, mL/min/1.73 m^2^	90.6 ± 17.7	92.0 ± 19.6	87.6 ± 19.8	81.2 ± 19.7	82.1 ± 18.0
Dosage of MTX, mg/week	10.2 ± 3.0	10.0 ± 2.9	10.1 ± 2.7	—	—
csDMARD use, n (%)	—	—	—	51 (54.3)	21 (45.7)
Glucocorticoid use, n (%)	37 (49.3)	62 (40.8)	64 (41.6)	49 (52.1)	21 (45.7)
Rheumatoid factor, IU/mL	112 ± 189	148 ± 267	145 ± 239	—	—
Seropositive RA, n (%)^a^	64 (85.3)	140 (92.1)	136 (88.3)	—	—
DAS28-CRP	5.1 ± 1.0	5.2 ± 1.1	5.1 ± 0.9	5.3 ± 1.0	5.2 ± 1.1
DAS28-ESR	5.8 ± 1.0	5.9 ± 1.0	5.8 ± 0.9	5.8 ± 1.0	5.8 ± 1.0
Tender joint count in 68 joints	15.5 ± 9.6	16.6 ± 8.8	15.6 ± 8.9	16.4 ± 9.8	14.1 ± 8.1
Swollen joint count in 66 joints	13.2 ± 8.5	13.8 ± 7.2	12.8 ± 6.4	12.1 ± 6.0	11.4 ± 6.7
hs-CRP, mg/dL	1.3 ± 1.7	1.6 ± 2.0	1.3 ± 1.8	2.1 ± 2.3	2.1 ± 2.2
ESR, mm/h	36.4 ± 17.3	40.3 ± 22.3	38.6 ± 20.6	41.1 ± 22.9	45.7 ± 28.4

Data are expressed as mean ± standard deviation in the full analysis set

*CRP* C-reactive protein, *csDMARD* conventional synthetic disease-modifying antirheumatic drug, *DAS28-CRP* Disease Activity Score in 28 joints based on CRP, *DAS28-ESR* Disease Activity Score in 28 joints based on ESR, *eGFR* estimated glomerular filtration rate, *ESR* erythrocyte sedimentation rate, *hs-CRP* high-sensitivity C-reactive protein, *MTX* methotrexate, *RA* rheumatoid arthritis

^a^Seropositive RA indicates an anti–cyclic citrullinated peptide antibody level ≥ 4.5 U/mL and/or rheumatoid factor level > 15 IU/mL

### PK profile and effects of patient baseline characteristics on plasma concentration

**Fig. 2:**
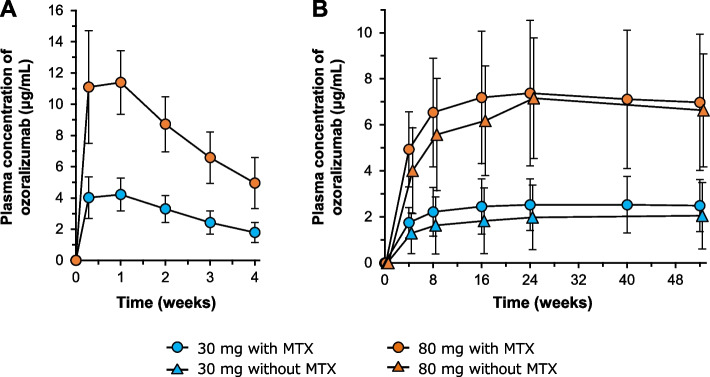
Plasma concentration–time profiles from the first dose to 4 weeks with MTX (**A**) and trough concentrations throughout 52 weeks with or without MTX (**B**). Data are expressed as mean ± standard deviation. *MTX* methotrexate

In the OHZORA trial, the plasma concentration of OZR reached the C_max_ at 144 h (median t_max_, 6 days) and decreased with a t_1/2_ of 17.9–18.2 days. The C_max_ and AUC increased in a dose-dependent manner (Table 2 and Fig. 2A). In both trials, the plasma OZR trough concentration reached a steady state by 16 weeks after the first administration (Fig. 2B). Trough concentrations at steady state in the 30 mg and 80 mg groups were approximately 2 μg/mL and 7 μg/mL, respectively. Analysis of the correlation coefficients for trough concentration at week 4, C_max_, and AUC_0-∞_ with patient baseline characteristics showed a negative correlation between these PK parameters and body weight. None of the other characteristics showed any significant correlation with these PK parameters (Additional file 1, Supplementary Table S1). Also, in the OHZORA trial, plasma OZR concentrations in patients with seropositive and seronegative RA were similar (Additional file 2, Supplementary Fig. S1).

**Table 2:** Pharmacokinetic parameters after a single subcutaneous administration of ozoralizumab

**Dose (mg)**	**N**	**C**_**max**_** (μg/mL)**	**t**_**max**_** (h)**	**AUC**_**0-last**_** (h·μg/mL)**	**AUC**_**0-∞**_** (h·μg/mL)**	**t**_**1/2**_** (day)**	**CL/F (mL/h)**	**V**_**d**_**/F (L)**
30	77	4.55 ± 1.18	144 (23.2–336)	2050 ± 523	3280 ± 1280^a^	18.2 ± 8.21^a^	10.4 ± 3.91^a^	5.88 ± 1.72^a^
80	80	12.5 ± 2.84	144 (22.1–212)	5540 ± 1080	8860 ± 3010	17.9 ± 5.98	9.92 ± 2.93	5.71 ± 1.09

Data are expressed as mean ± standard deviation or median (range)

*AUC* area under the plasma concentration–time curve, *AUC*_*0-∞*_ AUC from 0 to infinity, *AUC*_*0-last*_ AUC from 0 to the last quantifiable data point, *CL/F* apparent clearance, *C*_*max*_ maximum concentration, *t*_*1/2*_ elimination half-life, *t*_*max*_ time to maximum concentration, *V*_d_*/F* apparent volume of distribution

^a^*N* = 74

### Effects of patient baseline characteristics on ACR20 response

Regarding ACR20 response rates at week 16 in both trials, analyses stratified by patient baseline characteristics such as disease activity, presence/absence of concomitant use of MTX, and presence/absence of previous use of bDMARDs and TNFα inhibitors showed no effect of any of these characteristics on ACR20 response rates (Additional file 1, Supplementary Table S2).

### Effects of trough concentration on OZR efficacy

**Fig. 3:**
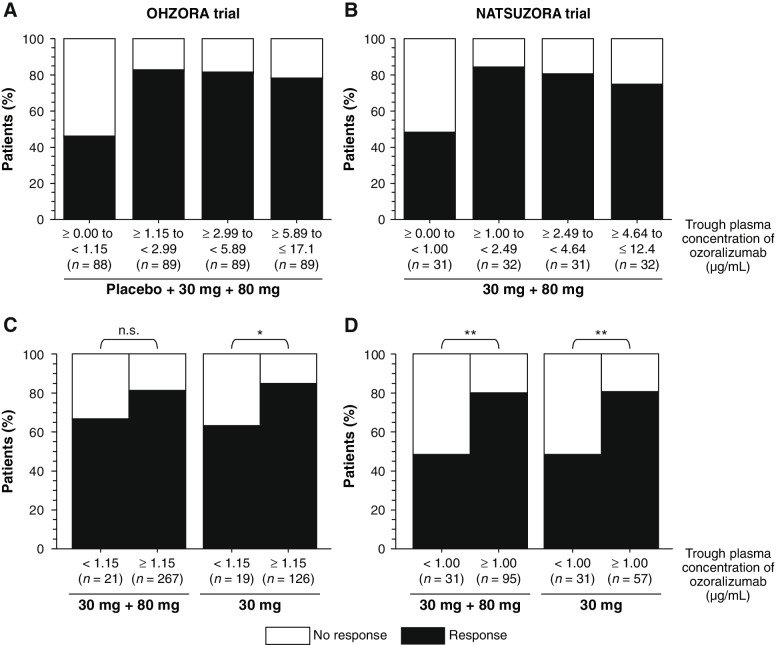
Association between trough plasma ozoralizumab concentration and ACR20 response at week 16. The 16-week ACR20 response rates were compared by quartile in the OHZORA trial (**A**) and the NATSUZORA trial (**B**), or stratified as above and below the boundary value, which was 1.15 µg/mL in the OHZORA trial (**C**) and 1.00 µg/mL in the NATSUZORA trial (**D**). Fisher’s exact test, *p < 0.05, **p < 0.01. *ACR20* ≥ 20% improvement according to the American College of Rheumatology criteria, *n.s.* not significant

In the OHZORA trial, ACR20 response rates were compared among four quartiles stratified by trough concentration at week 16: Q1, ≥ 0.00 to < 1.15 μg/mL; Q2, ≥ 1.15 to < 2.99 μg/mL; Q3, ≥ 2.99 to < 5.89 μg/mL; and Q4, ≥ 5.89 to ≤ 17.1 μg/mL. Of note, the ACR20 response rate was higher in the second, third, and fourth quartiles (Q2, Q3, and Q4), where the trough concentration was equal to or above the threshold (ie, 1.15 μg/mL), compared with the first quartile (Q1) (Fig. 3A). Moreover, comparison of ACR20 response rates stratified by < 1.15 vs ≥ 1.15 μg/mL in the OZR 30 mg and 80 mg groups together and the OZR 30 mg group alone showed a higher response rate in the higher trough subgroups compared with the lower trough subgroup (Fig. 3C). The NATSUZORA trial also showed the same trend with 1.00 μg/mL as the threshold value (Fig. 3B and D). In the 80 mg groups of the OHZORA and NATSUZORA trials, two patients and no patient, respectively, had a trough concentration below the threshold.

**Fig. 4:**
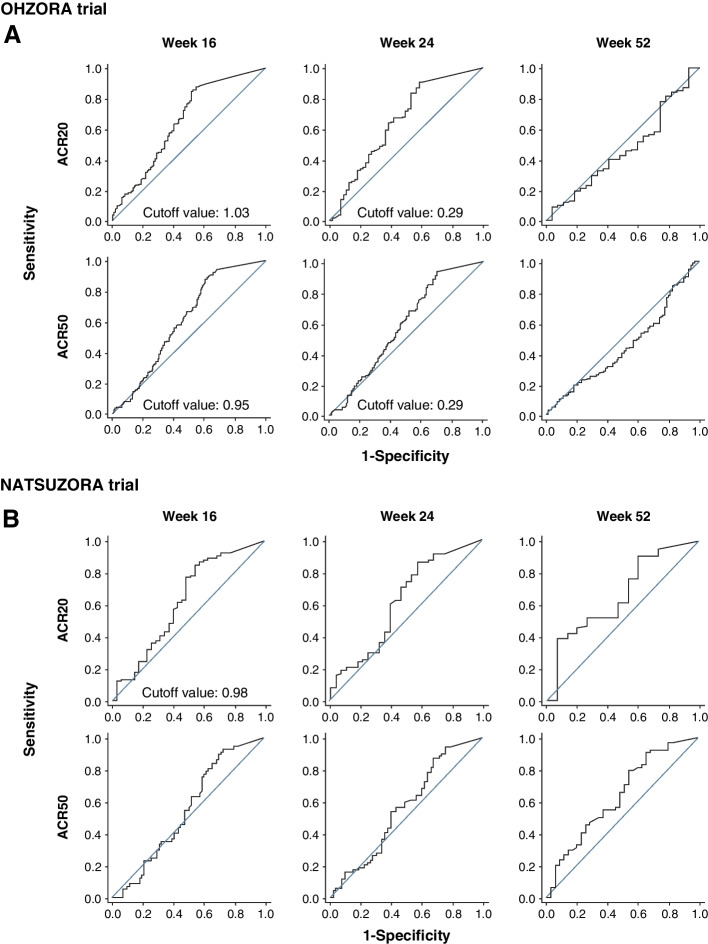
Association between trough plasma ozoralizumab concentration and ACR20 or ACR50 response in the OHZORA trial (**A**) and the NATSUZORA trial (**B**) using receiver operating characteristic analysis. *ACR20/50* ≥ 20%/50% improvement according to the American College of Rheumatology criteria

**Fig. 5:**
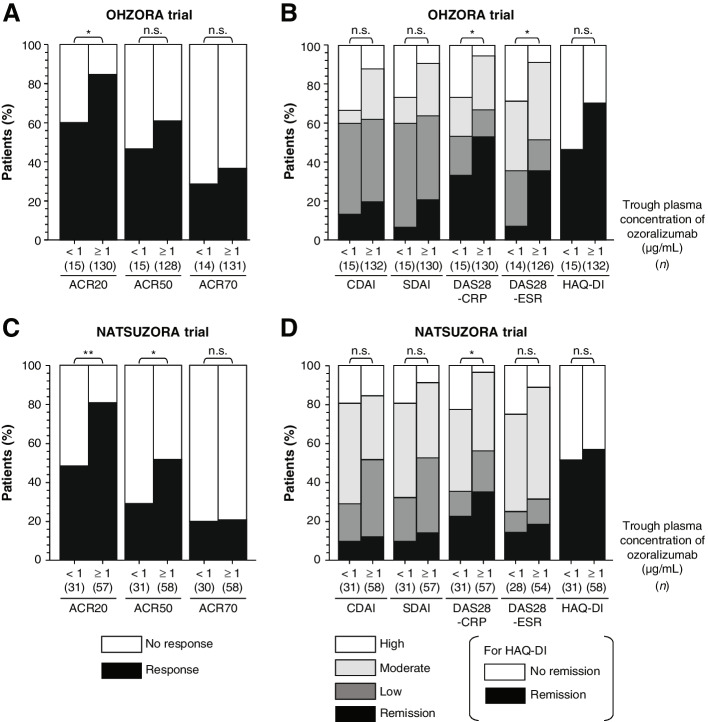
Efficacy stratified by trough concentration of ozoralizumab (1 μg/mL) at week 16 in the ozoralizumab 30 mg group in the OHZORA trial (**A**, **B**) and the NATSUZORA trial (**C**, **D**). No remission, HAQ-DI > 0.5; High, CDAI > 22, SDAI > 26, DAS28 > 5.1; moderate, 10 < CDAI ≤ 22, 11 < SDAI ≤ 26, 3.2 ≤ DAS28 ≤ 5.1; low, 2.8 < CDAI ≤ 10, 3.3 < SDAI ≤ 11, 2.6 ≤ DAS28 < 3.2; remission, CDAI ≤ 2.8, SDAI ≤ 3.3, DAS28 < 2.6, HAQ-DI ≤ 0.5. Fisher’s exact test, *p < 0.05, **p < 0.01. *ACR20/50/70* ≥ 20%/50%/70% improvement according to the American College of Rheumatology criteria, *CDAI* Clinical Disease Activity Index, *CRP* C-reactive protein, *DAS28-CRP* Disease Activity Score in 28 joints based on CRP, *DAS28-ESR* Disease Activity Score in 28 joints based on ESR, *ESR* erythrocyte sedimentation rate, *HAQ-DI* Health Assessment Questionnaire-Disability Index, *n.s.* not significant, *SDAI* Simplified Disease Activity Index

The ROC analysis of ACR20 or ACR50 response rates and trough concentrations is shown in Fig. 4. Cutoff values for ACR20 and ACR50 response rates in the OHZORA trial were 1.03 and 0.95 μg/mL, respectively, at week 16 and 0.29 μg/mL for both at week 24; no cutoff value was obtained at week 52 (Fig. 4A and Additional file 1, Supplementary Table S3). In the NATSUZORA trial, the cutoff value for the ACR20 response rate at week 16 was 0.98 μg/mL, and no cutoff value was obtained for the ACR50 response rate at week 16 or for the ACR20 or ACR50 response rates at weeks 24 or 52 (Fig. 4B and Additional file 1, Supplementary Table S3). On the basis of these results, when the 30 mg group at week 16 was stratified using the trough concentration in the OHZORA trial, ie, 1 μg/mL, the mean trough concentration (± standard deviation [SD]) for a subgroup with trough concentration equal to or above the cutoff value (higher trough subgroup) and a subgroup with trough concentration below the cutoff value (lower trough subgroup) was 2.63 ± 1.04 μg/mL (*n* = 132) and 0.45 ± 0.29 μg/mL (*n* = 15), respectively. When the 30 mg group at week 16 was stratified in the same manner in the NATSUZORA trial, ie, 1 μg/mL, the mean trough concentration for the higher trough subgroup and the lower trough subgroup was 2.67 ± 1.03 μg/mL (*n* = 58) and 0.27 ± 0.34 μg/mL (*n* = 31), respectively. In both trials, the mean concentrations in the higher trough subgroups were similar, and a 5.8- to 9.9-fold difference in mean concentration was observed between the higher and lower trough subgroups. Comparison of ACR20, ACR50, and 70% improvement according to the American College of Rheumatology criteria (ACR70) response rates between the higher and lower trough subgroups in the 30 mg groups of the OHZORA trial and NATSUZORA trial at week 16 showed that the response rates were significantly higher in the higher trough subgroup than in the lower trough subgroup for ACR20 (*p* < 0.05) in the OHZORA trial and for ACR20 (*p* < 0.01) and ACR50 (*p* < 0.05) in the NATSUZORA trial (Fig. 5A and C). A significant difference was observed between the higher and lower trough subgroups in DAS28-CRP (*p* < 0.05) and DAS28-ESR (*p* < 0.05) in the OHZORA trial and in DAS28-CPR (*p* < 0.05) in the NATSUZORA trial (Fig. 5B and D). Although there was no clear difference in the ACR20 response rate between the trials, the other improvement rates tended to be higher in the OHZORA trial, especially in the lower trough group (Fig. 5A-D).

### Effects of ADA/NAb on plasma concentration and ACR20 response

The proportion of patients whose ADA titers increased (TB-positive) or who became positive for ADA (TI-positive) between the start of OZR administration and week 52 was 30.8% (*n* = 44) and 29.2% (*n* = 45) in the OHZORA trial with OZR 30 mg and 80 mg, respectively [14], and 46.8% (*n* = 44) and 39.1% (*n* = 18) in the NATSUZORA trial with OZR 30 mg and 80 mg, respectively [15]. Comparison according to the status of ADA formation showed that the plasma concentration tended to decrease in the following order: negative > BL-positive > TB/TI-positive, in the 30 mg group of the NATSUZORA trial. However, the ACR20 response rate did not show a remarkable difference in either the OHZORA or the NATSUZORA trial (Additional file 3, Supplementary Fig. S2).

The proportion of NAb-positive patients by week 52 was 7.0% (*n* = 10) and 5.2% (*n* = 8) with OZR 30 mg and 80 mg in the OHZORA trial, respectively [14], and 27.7% (*n* = 26) and 2.2% (*n* = 1) with OZR 30 mg and 80 mg in the NATSUZORA trial, respectively [15]. Comparison according to the status of NAbs showed that the OZR plasma concentration decreased in NAb-positive patients in the 30 mg group of the NATSUZORA trial, and the ACR20 response rate at week 24 was 74.3% (55/74) in NAb-negative and 44.4% (8/18) in NAb-positive patients. Meanwhile, the ACR20 response rate at week 52 was 75.0% (51/68) and 68.0% (17/25) in NAb-negative and NAb-positive patients, respectively. As only two patients in the 30 mg group of the OHZORA trial at week 16 and one patient in the 80 mg group of the NATSUZORA trial at week 24 were NAb-positive, the effects of NAbs on OZR efficacy are unclear (Additional file 4, Supplementary Fig. S3) [15].

### Simulation of plasma OZR concentration

**Fig. 6:**
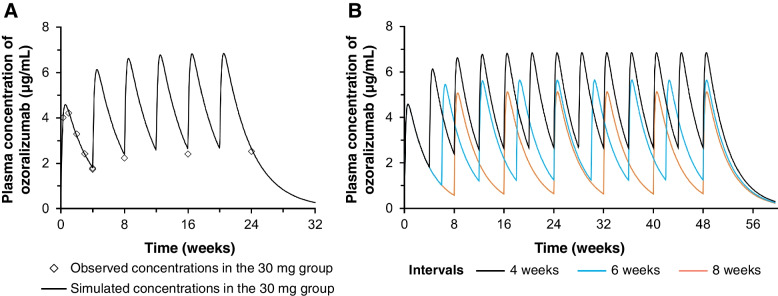
Simulation of plasma ozoralizumab concentration. Simulated values were compared with the observed values (**A**) and the concentrations after various dosing intervals were simulated (**B**)

By fitting the mean OZR plasma concentrations after the first administration in the 30 mg group of the OHZORA trial using a one-compartment model, the V_d_/F was estimated to be 5.53 L, K_a_ 0.0315 h^−1^, and CL/F 9.48 mL/h. These values were used to simulate plasma concentrations after repeated administration of OZR 30 mg at 4-week intervals. The results were compared with the observed values, and a good fit was confirmed (Fig. 6A). Simulation was also performed for the administration of OZR 30 mg at 4-, 6-, and 8-week intervals (Fig. 6B), and trough concentrations after reaching steady state were estimated to be 2.7, 1.2, and 0.6 μg/mL, respectively.

## Discussion

Our data showed a plasma half-life of 18 days and stable trough concentrations around 2 μg/mL after subcutaneous administration of OZR 30 mg. OZR lacks the fragment crystallizable (F_c_) portion and is therefore associated with reduced cytotoxicity; the prolonged retention in the blood has no added benefit owing to the neonatal F_c_ receptor–mediated recycling [17, 18]. However, addition of an albumin-binding domain is an established strategy to extend the plasma t_1/2_ of small antibody drugs and NANOBODY^®^ VHHs lacking the F_c_ portion [19, 20]. A study in mice has shown that the addition of an HSA-binding domain to a bivalent anti-TNFα NANOBODY^®^ VHH, albeit with a different amino acid sequence to that of OZR, extended its t_1/2_ from 54 min to 2.2 days after intravenous administration [9]. In cynomolgus monkeys, the addition of an HSA-binding domain reduced clearance after intravenous administration to less than 160-fold compared with an anti-interleukin (IL)-6 receptor NANOBODY^®^ VHH [21]. Moreover, the t_1/2_ of caplacizumab, a NANOBODY^®^ VHH without an HSA-binding domain, is 38.5 h in humans after subcutaneous administration [22], while an anti-IL-17 NANOBODY^®^ VHH compound with HSA-binding domains has a t_1/2_ of 11–12 days following subcutaneous administration in patients with psoriasis [23] and the t_1/2_ of OZR, which possesses an HSA-binding domain, is 18 days. These observations suggest that OZR has a long t_1/2_ because of its ability to bind HSA. Notably, no correlation was observed between the PK parameters of OZR and serum albumin levels. Since the molar ratio of serum albumin and OZR concentration is several thousand-fold, the effects of clinically low albumin concentrations on PK are thought to be limited.

A negative correlation was observed between body weight and PK parameters such as trough concentration, C_max_, AUC_0-∞_ in both trials, possibly because of increases in volume of distribution and clearance due to an increase in body weight. However, body weight did not affect the ACR20 response rate. It should be noted that the patients in both trials were all Japanese and had a lower body weight than that seen in patients with RA from other populations around the world. Further evaluation of the effects of patient demographic characteristics, including body weight, on PK is under consideration in a population PK analysis.

With regard to ADAs against OZR, 29.2%–46.8% of the patients showed an increase in antibody titer or newly generated antibodies by week 52 regardless of the dose of OZR administered and whether MTX was used concomitantly or not [14, 15]. But the effects on PK were modest at 30 mg without MTX, and they did not remarkably affect the ACR20 response rate. On the other hand, NAbs were observed in 27.7% of the patients in the 30 mg group without concomitant use of MTX by week 52 and in not greater than 7.0% of the patients in the other groups of both trials. As previously reported, the expression rate of NAbs increased in the absence of MTX and decreased with higher doses of the drug, an effect thought to be general to anti-TNF antibodies [15]. The improvement rates other than ACR20 tended to be lower without MTX, possibly due to the decrease in trough concentration associated with the generation of NAbs when MTX was not used.

The efficacy of TNF inhibitors decreases when the trough concentration decreases due to the trough concentration falling below the lower limit of the therapeutic window [24–27]. The plasma concentrations of OZR in Japanese patients with RA increased in a dose-dependent manner from 30 to 80 mg, while the ACR20 response rate was similar for 30 mg and 80 mg [10, 14, 15]. However, the results of the ROC analysis of the relationship between trough concentrations and ACR20 or ACR50 response rates showed a cutoff value of 1 μg/mL at week 16 in patients with and without MTX. Higher efficacy was observed in the higher trough group (≥ the cutoff value). Subsequently, the cutoff value decreased or could not be determined at week 24 and was not achieved at week 52. These results indicate that efficacy can be expected with long-term administration independent of trough concentration and that 30 mg of OZR is an adequate dose to maintain OZR concentration within the therapeutic window. Moreover, when 30 mg was administered at 6-week intervals, the trough concentration was estimated by PK simulation to be 1.2 μg/mL, which exceeds the cutoff value obtained in this study. Therefore, efficacy can be expected even if the administration interval is extended to 6 weeks.

The present study has some limitations. Post hoc analyses including ROC were not preplanned. The number of patients with low trough concentrations in the 30 mg group in the OHZORA trial may not have been sufficient for the stratified analysis. For the ROC analysis, the ACR20 response, the primary endpoint, and the ACR50 response, the secondary endpoint for progression of the disease after the ACR20 response, were selected, but analyses using other endpoints may differ.

## Conclusions

The results of the present study suggest that OZR has a long t_1/2_ and good PK properties that are unlikely to be influenced by patient background characteristics except for body weight. The efficacy of OZR was investigated on the basis of PK, and the results showed that subcutaneous administration of OZR 30 mg at 4-week intervals, which is the recommended clinical dosage and administration frequency of OZR, was more effective when the trough concentration at week 16 was more than 1 μg/mL. At week 52, however, efficacy was not dependent on the trough concentration. Therefore, we suggest that the efficacy with long-term administration, such as 52 weeks, is independent of the trough concentration.

## Supplementary Information

**Additional file 1: Supplementary Table S1.** Relationship between patient baseline characteristics and pharmacokinetic parameters of ozoralizumab. **Supplementary Table S2.** ACR20 response stratified by patient baseline characteristics. **Supplementary Table S3.** ROC analysis of ACR20 or ACR50 response and trough plasma ozoralizumab concentrations.

**Additional file 2: Supplementary Fig. S1.** Comparison of plasma ozoralizumab concentrations in patients with seropositive and seronegative RA. Plasma concentration–time profiles from the first dose to 4 weeks (A, B) and trough concentrations throughout 52 weeks (C, D) with methotrexate are shown. Seropositive RA indicates an anti–cyclic citrullinated peptide antibody level ≥ 4.5 U/mL and/or rheumatoid factor level > 15 IU/mL. *RA *rheumatoid arthritis, *SD* standard deviation.

**Additional file 3: Supplementary Fig. S2.** Effects of anti-drug antibodies on plasma ozoralizumab concentration and ACR20 response. *ACR20* ≥ 20% improvement according to the American College of Rheumatology criteria, *BL* baseline, *SD* standard deviation, *TB* treatment-boosted, *TI* treatment-induced.

**Additional file 4: Supplementary Fig. S3.** Effects of neutralizing antibodies on plasma ozoralizumab concentration and ACR20 response. ^a^Trough concentrations of the neutralizing antibody–positive patients in the 30 mg group were not calculated because a large number of the measurements were below the limit of quantification. ^b^Trough concentrations of the neutralizing antibody–positive patients in the 80 mg group are not shown as only one patient in the 80 mg group was neutralizing antibody–positive by the end of week 8. *ACR20* ≥ 20% improvement according to the American College of Rheumatology criteria, *SD* standard deviation.

## Data Availability

Data generated and/or analyzed in the current study were provided by Taisho Pharmaceutical Co., Ltd. under license and cannot be made freely available. Requests for access to these data should be made to the corresponding author.

## Abbreviations

ACR: American College of Rheumatology
ACR20/50/70: ≥ 20%/50%/70% Improvement according to the ACR criteria
ADA: Anti-drug antibody
AUC: Area under the curve
AUC_0-∞_: AUC from 0 to infinity
bDMARD: Biologic disease-modifying antirheumatic drug
BL: Baseline
CDAI: Clinical Disease Activity Index
CI: Confidence interval
CL/F: Apparent clearance
C_max_: Maximum concentration
CRP: C-reactive protein
csDMARD: Conventional synthetic disease-modifying antirheumatic drug
DAS: Disease Activity Score
DAS28-CRP: Disease Activity Score in 28 joints based on CRP
DAS28-ESR: Disease Activity Score in 28 joints based on ESR
EE: Early escape
ELISA: Enzyme-linked immunosorbent assay
ESR: Erythrocyte sedimentation rate
FAS: Full analysis set
F_c_: Fragment crystallizable
HSA: Human serum albumin
hs-CRP: High-sensitivity C-reactive protein
IL: Interleukin
JAK: Janus-associated kinase
K_a_: Association constant
MTX: Methotrexate
NAb: Neutralizing antibody
NATSUZORA: Efficacy and safety of anti-TNF alpha multivalent NANOBODY^®^ compound ozoralizumab in patients with rheumatoid arthritis and an inadequate response to methotrexate in a randomized, double-blind, placebo-controlled trial
OHZORA: Efficacy and safety of anti-TNF alpha multivalent NANOBODY^®^ compound ozoralizumab in patients with active rheumatoid arthritis, without methotrexate co-administration in a randomized open-label trial
OZR: Ozoralizumab
PK: Pharmacokinetics
RA: Rheumatoid arthritis
ROC: Receiver operating characteristic
SD: Standard deviation
SDAI: Simplified Disease Activity Index
SJC66: Swollen joint count in 66 joints
t_1/2_: Half-life
TB: Treatment-boosted
TI: Treatment-induced
TJC68: Tender joint count in 68 joints
t_max_: Time to C_max_
TNF: Tumor necrosis factor
TNFα: Tumor necrosis factor alpha
V_d_/F: Apparent volume of distribution
VHH: Variable region of heavy-chain-only antibody
