# Supplementary material for: Efficacy and pharmacokinetics of ozoralizumab, an anti-TNFα NANOBODY® compound, in patients with rheumatoid arthritis: 52-week results from the OHZORA and NATSUZORA trials
Source: Arthritis Res Ther. 2023 Apr 13;25:60. doi: 10.1186/s13075-023-03036-4 (PMC10099673; doi:10.1186/s13075-023-03036-4)
Supplement: Supplementary file 1 — Additional file 1: Supplementary Table S1. Relationship between patient baseline characteristics and pharmacokinetic parameters of ozoralizumab. Supplementary Table S2. ACR20 response stratified by patient baseline characteristics. Supplementary Table S3. ROC analysis of ACR20 or ACR50 response and trough plasma ozoralizumab concentrations. [file 13075_2023_3036_MOESM1_ESM.docx]

**Supplementary Table S1** Relationship between patient baseline characteristics and pharmacokinetic parameters of ozoralizumab

| **Characteristic** | **OHZORA trial** | | | | | | **NATSUZORA trial** | |
| --- | --- | --- | --- | --- | --- | --- | --- | --- |
|  | **Ozoralizumab  30 mg + MTX** | | | **Ozoralizumab  80 mg + MTX** | | | **Ozoralizumab  30 mg** | **Ozoralizumab  80 mg** |
|  | **C_trough_**  **(μg/mL)** | **C_max_**  **(μg/mL)** | **AUC_0-∞_**  **(h·μg/mL)** | **C_trough_**  **(μg/mL)** | **C_max_**  **(μg/mL)** | **AUC_0-∞_**  **(h·μg/mL)** | **C_trough_**  **(μg/mL)** | **C_trough_**  **(μg/mL)** |
| Age, years | 0.0959 | -0.203 | 0.0586 | 0.204 | -0.121 | 0.0448 | 0.193 | 0.0957 |
| Body weight, kg | -0.519 | -0.603 | -0.374 | -0.638 | -0.498 | -0.500 | -0.435 | -0.321 |
| Disease duration, years | 0.0826 | 0.124 | -0.0818 | 0.0761 | -0.00133 | 0.0592 | 0.0532 | 0.197 |
| Serum albumin, g/dL | 0.158 | 0.116 | 0.247 | 0.0505 | 0.00106 | 0.100 | -0.0693 | 0.122 |
| eGFR, mL/min/1.73 m^2^ | -0.0926 | -0.0326 | -0.123 | -0.121 | -0.0575 | -0.0833 | -0.315 | -0.120 |
| Dosage of MTX, mg/week | -0.0642 | -0.166 | 0.0237 | -0.204 | -0.0856 | -0.133 | ― | ― |
| Rheumatoid factor, IU/mL | -0.165 | -0.0869 | -0.0144 | -0.0560 | -0.00540 | -0.0541 | ― | ― |
| DAS28-CRP | -0.172 | -0.117 | -0.0366 | 0.0366 | -0.00197 | -0.0372 | -0.0359 | -0.484 |
| DAS28-ESR | -0.102 | -0.0436 | 0.0790 | 0.208 | 0.155 | 0.159 | 0.0789 | -0.381 |
| Tender joint count in 68 joints | -0.0366 | -0.0109 | 0.0462 | 0.150 | 0.0898 | 0.161 | 0.192 | -0.292 |
| Swollen joint count in 66 joints | -0.120 | 0.0554 | 0.0000851 | 0.0496 | 0.0991 | 0.109 | 0.0226 | -0.321 |
| Pt-PA, mm | -0.0841 | -0.0152 | 0.0364 | 0.0348 | -0.153 | 0.00299 | -0.111 | -0.191 |
| Ph-GA, mm | -0.185 | -0.0884 | -0.194 | -0.00353 | -0.108 | -0.0273 | -0.149 | -0.154 |
| Pt-GA, mm | -0.140 | -0.0407 | 0.00498 | -0.00636 | -0.178 | -0.109 | -0.0573 | -0.294 |
| CDAI | -0.110 | -0.104 | 0.000244 | 0.0906 | 0.0394 | 0.0575 | 0.0333 | -0.413 |
| SDAI | -0.139 | -0.111 | -0.0243 | 0.0595 | 0.0385 | 0.0380 | -0.00947 | -0.460 |
| HAQ-DI | -0.122 | -0.0701 | -0.0328 | 0.0512 | 0.0876 | -0.0702 | 0.162 | -0.266 |
| mTSS | 0.0234 | -0.00157 | -0.0632 | 0.156 | 0.0125 | 0.123 | ― | ― |
| Erosion score | 0.0636 | 0.0142 | -0.0202 | 0.169 | -0.00481 | 0.109 | ― | ― |
| JSN score | -0.0209 | -0.0179 | -0.102 | 0.136 | 0.0294 | 0.132 | ― | ― |
| hs-CRP, mg/dL | -0.235 | -0.0743 | -0.192 | -0.103 | -0.00107 | -0.151 | -0.153 | -0.353 |
| ESR, mm/h | -0.136 | -0.00563 | -0.0182 | 0.261 | 0.261 | 0.247 | -0.0286 | -0.109 |

Data are expressed as regression coefficient, r

*AUC_0-∞_* area under the plasma concentration–time curve from 0 to infinity, *C_max_* maximum plasma concentration, *C_trough_* trough plasma concentration, *CDAI* Clinical Disease Activity Index, *CRP* C-reactive protein, *DAS* Disease Activity Score, *DAS28-CRP* Disease Activity Score in 28 joints based on CRP, *DAS28-ESR* Disease Activity Score in 28 joints based on ESR, *eGFR* estimated glomerular filtration rate, *ESR* erythrocyte sedimentation rate, *HAQ-DI* Health Assessment Questionnaire-Disability Index, *hs-CRP* high-sensitivity CRP, *JSN* joint space narrowing, *mTSS* modified Total Sharp Score, *MTX* methotrexate, *Ph-GA* physician’s global assessment of disease activity, *Pt-GA* patient’s global assessment of disease activity, *Pt-PA* patient’s assessment of pain, *SDAI* Simplified Disease Activity Index

**Supplementary Table S2** ACR20 response stratified by patient baseline characteristics

| **Characteristic** | **Subgroup** | **OHZORA trial** | | | **NATSUZORA trial** | |
| --- | --- | --- | --- | --- | --- | --- |
|  |  | **ACR20 response at week 16, % (*n*/*N*)** | | | **ACR20 response at week 24, % (*n*/*N*)** | |
|  |  | **Placebo + MTX** | **Ozoralizumab 30 mg + MTX** | **Ozoralizumab  80 mg + MTX** | **Ozoralizumab  30 mg** | **Ozoralizumab  80 mg** |
| Age, years | < 65 | 34.5 (19/55) | 77.1 (91/118) | 78.3 (90/115) | 66.7 (38/57) | 74.1 (20/27) |
|  | ≥ 65 | 47.4 (9/19) | 93.8 (30/32) | 70.3 (26/37) | 71.4 (25/35) | 72.2 (13/18) |
| Gender | Female | 35.7 (20/56) | 77.9 (81/104) | 74.4 (90/121) | 66.7 (46/69) | 69.2 (27/39) |
|  | Male | 44.4 (8/18) | 87.0 (40/46) | 83.9 (26/31) | 73.9 (17/23) | 100.0 (6/6) |
| Body weight, kg | < 40 | 0.0 (0/4) | 100.0 (5/5) | 100.0 (4/4) | 0.0 (0/1) | 0.0 (0/0) |
|  | ≥ 40 to < 50 | 25.0 (4/16) | 77.8 (21/27) | 77.5 (31/40) | 59.3 (16/27) | 100.0 (10/10) |
|  | ≥ 50 to < 60 | 37.0 (10/27) | 69.8 (37/53) | 68.5 (37/54) | 85.7 (24/28) | 47.4 (9/19) |
|  | ≥ 60 to < 70 | 41.2 (7/17) | 87.9 (29/33) | 81.5 (22/27) | 66.7 (12/18) | 90.9 (10/11) |
|  | ≥ 70 | 70.0 (7/10) | 90.6 (29/32) | 81.5 (22/27) | 61.1 (11/18) | 80.0 (4/5) |
| Seropositivity^a^ | Positive | 39.1 (25/64) | 79.7 (110/138) | 76.9 (103/134) | ― | ― |
|  | Negative | 30.0 (3/10) | 91.7 (11/12) | 76.5 (13/17) | ― | ― |
| DAS28-CRP | < 3.2 | 100.0 (1/1) | 100.0 (1/1) | 0.0 (0/0) | 0.0 (0/0) | 66.7 (2/3) |
|  | ≥ 3.2 to ≤ 5.1 | 40.6 (13/32) | 81.9 (59/72) | 78.3 (65/83) | 81.6 (31/38) | 70.6 (12/17) |
|  | > 5.1 | 36.8 (14/38) | 79.2 (61/77) | 75.0 (51/68) | 60.4 (32/53) | 76.0 (19/25) |
| DAS28-ESR | < 3.2 | 100.0 (1/1) | 0.0 (0/0) | 0.0 (0/0) | 0.0 (0/0) | 0.0 (0/0) |
|  | ≥ 3.2 to ≤ 5.1 | 36.8 (7/19) | 84.4 (27/32) | 72.0 (18/25) | 77.3 (17/22) | 75.0 (9/12) |
|  | > 5.1 | 37.7 (20/53) | 82.7 (91/110) | 78.0 (96/123) | 67.7 (44/65) | 71.0 (22/31) |
| CDAI | ≤ 10.0 | 0.0 (0/0) | 0.0 (0/0) | 0.0 (0/0) | 0.0 (0/0) | 0.0 (0/0) |
|  | > 10.0 to ≤ 22.0 | 64.7 (11/17) | 80.8 (21/26) | 76.0 (19/25) | 77.8 (14/18) | 66.7 (8/12) |
|  | > 22.0 | 30.4 (17/56) | 80.5 (99/123) | 75.8 (94/124) | 66.2 (49/74) | 75.8 (25/33) |
| SDAI | ≤ 11.0 | 0.0 (0/0) | 0.0 (0/0) | 0.0 (0/0) | 0.0 (0/0) | 0.0 (0/0) |
|  | > 11.0 to ≤ 26.0 | 52.2 (12/23) | 81.0 (34/42) | 76.2 (32/42) | 78.3 (18/23) | 68.8 (11/16) |
|  | > 26.0 | 33.3 (16/48) | 80.4 (86/107) | 76.4 (81/106) | 66.2 (45/68) | 75.9 (22/29) |
| hs-CRP, mg/dL | < 1.0 | 50.0 (23/46) | 85.7 (72/84) | 78.5 (73/93) | 71.4 (25/35) | 80.0 (16/20) |
|  | ≥ 1.0 | 19.2 (5/26) | 74.2 (49/66) | 74.1 (43/58) | 67.9 (38/56) | 68.0 (17/25) |
| Prior biologic DMARD use | No | 37.8 (17/45) | 76.8 (73/95) | 73.9 (68/92) | ― | ― |
|  | Yes | 37.9 (11/29) | 87.3 (48/55) | 80.0 (48/60) | ― | ― |
| Prior number of TNF inhibitors used | None | 35.8 (19/53) | 77.1 (81/105) | 74.8 (80/107) | ― | ― |
|  | 1 | 42.9 (6/14) | 86.7 (26/30) | 77.4 (24/31) | ― | ― |
|  | ≥ 2 | 42.9 (3/7) | 93.3 (14/15) | 85.7 (12/14) | ― | ― |

^a^Seropositive rheumatoid arthritis indicates an anti–cyclic citrullinated peptide antibody level ≥ 4.5 U/mL and/or rheumatoid factor level > 15 IU/mL

*ACR20* ≥ 20% improvement according to the American College of Rheumatology criteria, *CDAI* Clinical Disease Activity Index, *CRP* C-reactive protein, *DAS28-CRP* Disease Activity Score in 28 joints based on CRP, *DAS28-ESR* Disease Activity Score in 28 joints based on ESR, *DMARD* disease-modifying antirheumatic drug, *ESR* erythrocyte sedimentation rate, *hs-CRP* high-sensitivity CRP, *MTX* methotrexate, *SDAI* Simplified Disease Activity Index, *TNF* tumor necrosis factor

**Supplementary Table S3** ROC analysis of ACR20 or ACR50 response and trough plasma ozoralizumab concentrations

| **ROC analysis** | | **AUC** | | | **Lower limit of  95% CI of AUC** | | | **Cutoff value** | | | **Sensitivity at cutoff value** | | |
| --- | --- | --- | --- | --- | --- | --- | --- | --- | --- | --- | --- | --- | --- |
|  |  | **16W** | **24W** | **52W** | **16W** | **24W** | **52W** | **16W** | **24W** | **52W** | **16W** | **24W** | **52W** |
| OHZORA trial | ACR20 | 0.65 | 0.65 | 0.46 | 0.58 | 0.57 | 0.35 | 1.0 | 0.29 | 0.066 | 0.85 | 0.91 | 1.0 |
|  | ACR50 | 0.61 | 0.58 | 0.45 | 0.55 | 0.51 | 0.38 | 0.95 | 0.29 | 0.23 | 0.91 | 0.94 | 0.99 |
| NATSUZORA trial | ACR20 | 0.62 | 0.62 | 0.66 | 0.50 | 0.49 | 0.51 | 0.98 | 1.2 | 3.9 | 0.85 | 0.86 | 0.39 |
|  | ACR50 | 0.54 | 0.56 | 0.64 | 0.44 | 0.45 | 0.52 | 0.52 | 1.2 | 1.8 | 0.93 | 0.87 | 0.797 |

*ACR20/50* ≥ 20%/50% improvement according to the American College of Rheumatology criteria, *AUC* area under the ROC curve, *CI* confidence interval, *ROC* receiver operating characteristic, *W* week
