# Supplementary material for: Efficacy and pharmacokinetics of ozoralizumab, an anti-TNFα NANOBODY® compound, in patients with rheumatoid arthritis: 52-week results from the OHZORA and NATSUZORA trials
Source: Arthritis Res Ther. 2023 Apr 13;25:60. doi: 10.1186/s13075-023-03036-4 (PMC10099673; doi:10.1186/s13075-023-03036-4)

Supplementary Fig. S1. Comparison of plasma ozoralizumab concentrations in patients with seropositive and seronegative RA. Plasma concentration–time profiles from the first dose to 4 weeks (A, B) and trough concentrations throughout 52 weeks (C, D) with methotrexate are shown. Seropositive RA indicates an anti–cyclic citrullinated peptide antibody level ≥ 4.5 U/mL and/or rheumatoid factor level > 15 IU/mL. *RA* rheumatoid arthritis, *SD* standard deviation.

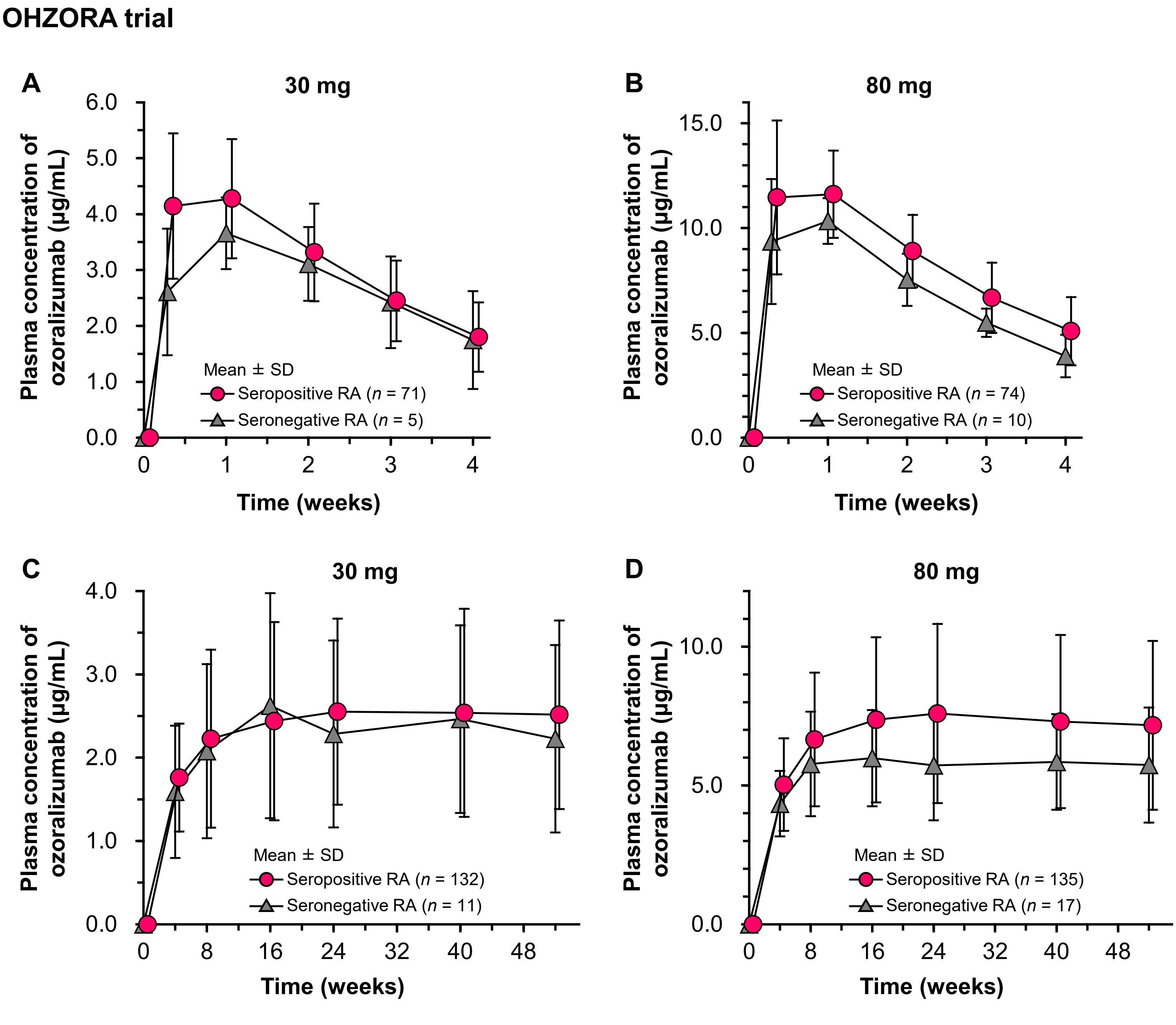

Supplement: Supplementary file 2 — Additional file 2: Supplementary Fig. S1. Comparison of plasma ozoralizumab concentrations in patients with seropositive and seronegative RA. Plasma concentration–time profiles from the first dose to 4 weeks (A, B) and trough concentrations throughout 52 weeks (C, D) with methotrexate are shown. Seropositive RA indicates an anti–cyclic citrullinated peptide antibody level ≥ 4.5 U/mL and/or rheumatoid factor level > 15 IU/mL. RA rheumatoid arthritis, SD standard deviation. [file 13075_2023_3036_MOESM2_ESM.pdf]
