# Supplementary material for: Efficacy and pharmacokinetics of ozoralizumab, an anti-TNFα NANOBODY® compound, in patients with rheumatoid arthritis: 52-week results from the OHZORA and NATSUZORA trials
Source: Arthritis Res Ther. 2023 Apr 13;25:60. doi: 10.1186/s13075-023-03036-4 (PMC10099673; doi:10.1186/s13075-023-03036-4)

Supplementary Fig. S2. Effects of anti-drug antibodies on plasma ozoralizumab concentration and ACR20 response.
ACR20 ≥ 20% improvement according to the American College of Rheumatology criteria, *BL* baseline, *SD* standard deviation, *TB* treatment-boosted, *TI* treatment-induced.

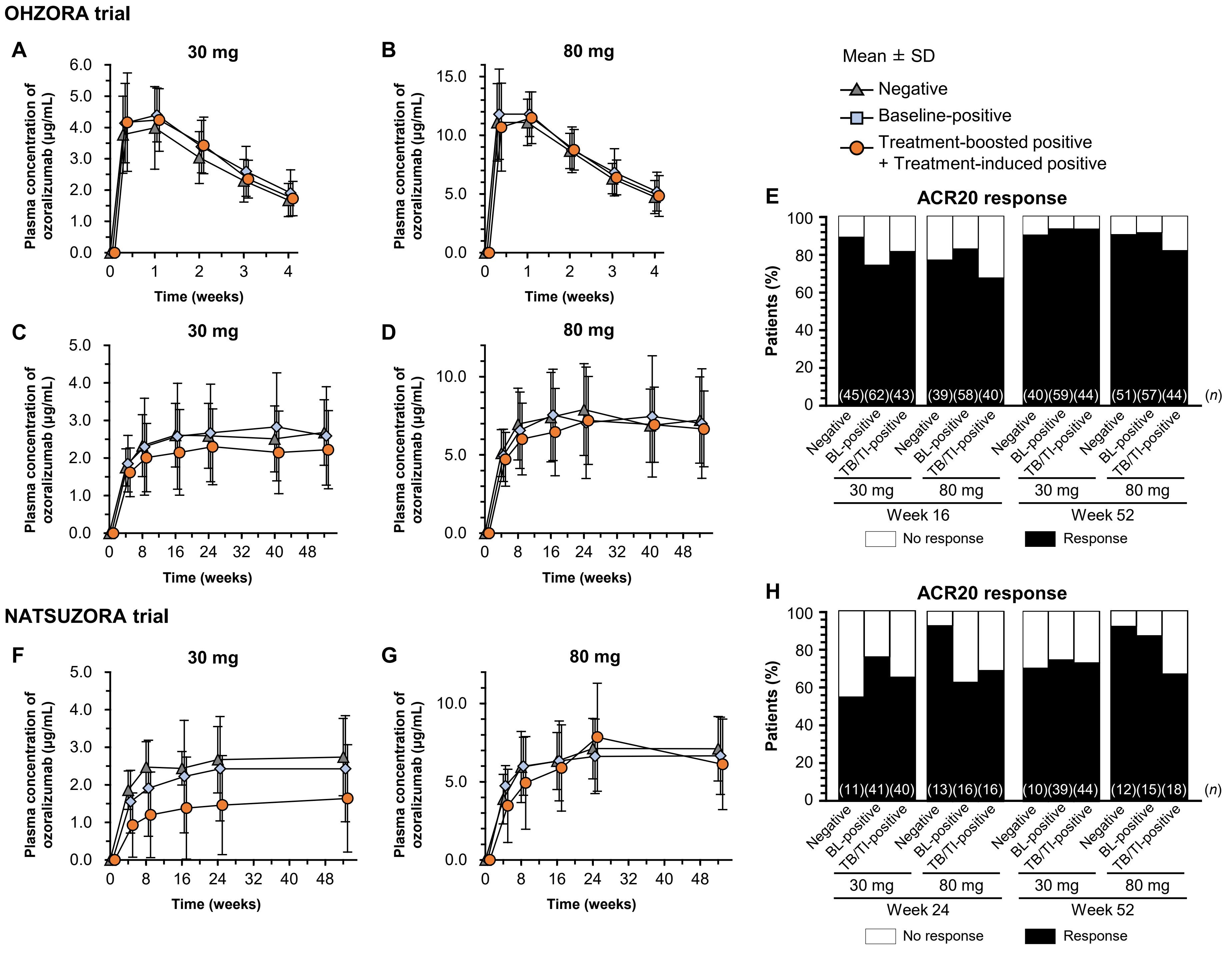

Supplement: Supplementary file 3 — Additional file 3: Supplementary Fig. S2. Effects of anti-drug antibodies on plasma ozoralizumab concentration and ACR20 response. ACR20 ≥ 20% improvement according to the American College of Rheumatology criteria, BL baseline, SD standard deviation, TB treatment-boosted, TI treatment-induced. [file 13075_2023_3036_MOESM3_ESM.pdf]
