# Supplementary material for: Efficacy and pharmacokinetics of ozoralizumab, an anti-TNFα NANOBODY® compound, in patients with rheumatoid arthritis: 52-week results from the OHZORA and NATSUZORA trials
Source: Arthritis Res Ther. 2023 Apr 13;25:60. doi: 10.1186/s13075-023-03036-4 (PMC10099673; doi:10.1186/s13075-023-03036-4)

Supplementary Fig. S3. Effects of neutralizing antibodies on plasma ozoralizumab concentration and ACR20 response.

[a] Trough concentrations of the neutralizing antibody–positive patients in the 30 mg group were not calculated because a large number of the measurements were below the limit of quantification.

[b] Trough concentrations of the neutralizing antibody–positive patients in the 80 mg group are not shown as only one patient in the 80 mg group was neutralizing antibody–positive by week 8.

ACR20 ≥ 20% improvement according to the American College of Rheumatology criteria, *SD* standard deviation.

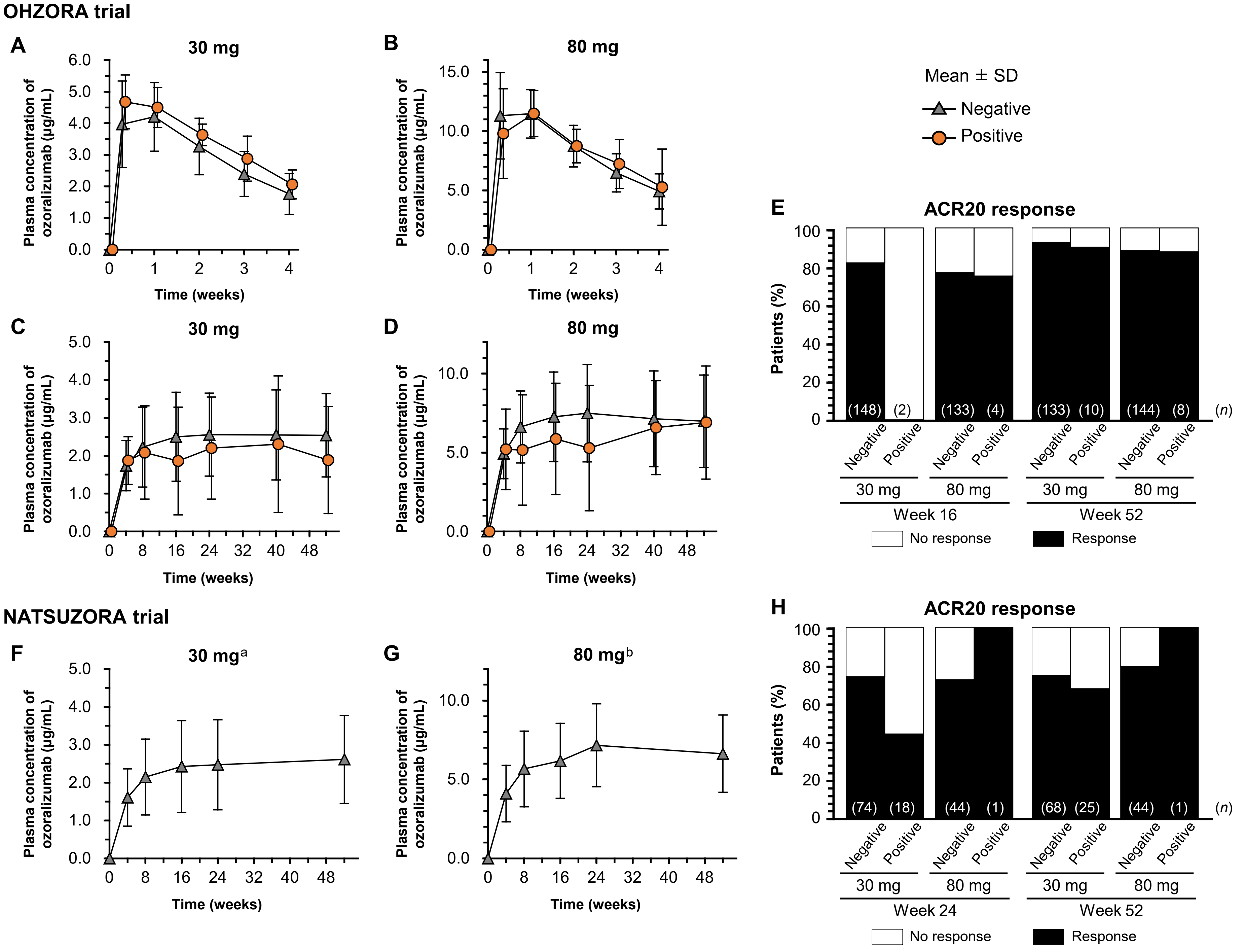

Supplement: Supplementary file 4 — Additional file 4: Supplementary Fig. S3. Effects of neutralizing antibodies on plasma ozoralizumab concentration and ACR20 response. aTrough concentrations of the neutralizing antibody–positive patients in the 30 mg group were not calculated because a large number of the measurements were below the limit of quantification. bTrough concentrations of the neutralizing antibody–positive patients in the 80 mg group are not shown as only one patient in the 80 mg group was neutralizing antibody–positive by the end of week 8. ACR20 ≥ 20% improvement according to the American College of Rheumatology criteria, SD standard deviation. [file 13075_2023_3036_MOESM4_ESM.pdf]
